# The Real-World Clinical Outcomes of Favipiravir Treatment with Telemedicine Monitoring in Preventing Disease Progression in Mild to Moderate COVID-19 Patients; A Retrospective Cohort Study

**DOI:** 10.3390/medicina59061098

**Published:** 2023-06-06

**Authors:** Taweegrit Siripongboonsitti, Kriangkrai Tawinprai, Kunsuda Cheirsilpa, Teerapat Ungtrakul, Wasanai Krisorakun, Chanisa Chotipanich, Nat Wimolsiri, Permpen Noitun, Netnapis Srirattana, Nithi Mahanonda

**Affiliations:** 1 Division of Infectious Diseases, Department of Medicine, Chulabhorn Hospital, Chulabhorn Royal Academy, Bangkok 10210, Thailand; kriangkrai.taw@cra.ac.th; 2 Princess Srisavangavadhana College of Medicine, Chulabhorn Royal Academy, Bangkok 10210, Thailand; 3 Division of Gastroenterology, Department of Medicine, Chulabhorn Hospital, Chulabhorn Royal Academy, Bangkok 10210, Thailand; kunsuda.che@cra.ac.th; 4 Department of Obstetrics & Gynecology, Chulabhorn Hospital, Chulabhorn Royal Academy, Bangkok 10210, Thailand; 5 National Cyclotron and PET Center, Chulabhorn Hospital, Chulabhorn Royal Academy, Bangkok 10210, Thailand; chanisa.cho@cra.ac.th; 6 Department of Radiology, Chulabhorn Hospital, Chulabhorn Royal Academy, Bangkok 10210, Thailand; nat.wim@cra.ac.th; 7 Cardiovascular Center, Chulabhorn Hospital, Chulabhorn Royal Academy, Bangkok 10210, Thailand; permpen.noi@cra.ac.th (P.N.); netnapis.sri@cra.ac.th (N.S.); 8 Chulabhorn Royal Academy, Bangkok 10210, Thailand; nithi.mah@cra.ac.th

**Keywords:** COVID-19, SARS-CoV-2, telemedicine, telehealth, favipiravir

## Abstract

*Background*: Favipiravir has complex pharmacokinetics, and varied efficacy has been reported in treating COVID-19. Telehealth and telemonitoring are disruptive challenges used for COVID-19 care during pandemics. *Objective*: This study aimed to assess the outcome of favipiravir treatment to prevent clinical deterioration in mild to moderate COVID-19 cases with adjunctive telemonitoring during the COVID-19 surge. *Methods*: This was a retrospective observational study of PCR-confirmed mild to moderate COVID-19 cases subjected to home isolation. Chest computed tomography (CT) was performed in all cases, and favipiravir was administrated. *Results*: This study involved 88 PCR-confirmed COVID-19 cases. In addition, 42/42 (100%) cases were Alpha variants. COVID-19 pneumonia was found in 71.5% of the cases, according to chest X-rays and chest CT on the first visit. Favipiravir started 4 days after symptoms, which was part of the standard of care. The 12.5% of the patients required supplemental oxygen and intensive care unit admission rate was 1.1%; 1.1% required mechanical ventilation, and the rate of all-cause mortality was 1.1%, with a value of 0% of severe COVID-19 deaths. All mild illness cases showed no clinical deterioration or requirement for supplemental oxygen. No significant deterioration in either obesity or diabetes mellitus was observed. *Conclusions*: Favipiravir treatment for mild to moderate COVID-19 cases in outpatient settings, coupled with telemonitoring, was both safe and effective in preventing clinical deterioration, including the need for oxygen supplementation. This approach proved valuable during surges of COVID-19 cases.

## 1. Introduction

The coronavirus disease 2019 (COVID-19) pandemic is caused by severe acute respiratory syndrome coronavirus-2 (SARS-CoV-2) and is associated with high mortality, with a 2.3% case fatality rate [1,2]. The cumulative global COVID-19 death cases were 6.6 million as of November 2022 [3]. Although remdesivir showed rapidly improved clinical status in hospitalized moderate COVID-19 patients, the intravenous agent use was limited [4,5]. COVID-19 treatment strategies focus on antiviral treatment to inhibit viral replication in the early phase to prevent disease progression [6]. Although many novel agents’ treatment provides good efficacy but still have some challenges; ritonavir-boosted nirmatrelvir has many drug interactions, and molnupiravir is associated with mutagenesis in mammalian cells [7]. The benefit of COVID-19 monoclonal antibodies decreased when a new variant of concern emerged.

The global scarcity of medical resources was a significant health system problem. Teleconsultation, telemonitoring, and outpatient clinic visits for COVID-19 patients have been mentioned rarely [8,9]. 

Favipiravir, a purine nucleic acid analog, is a broad-spectrum RNA-dependent RNA polymerase (RdRp) inhibitor of RNA viruses, including SARS-CoV-2, Ebola, and influenza [10,11]. A study in hamsters demonstrated that favipiravir decreased viral infectivity [12]. Favipiravir was mainly used in China, Russia, Japan, and India and used in first-line drug COVID-19 treatment in Thailand [13,14,15]. Recent studies showed that favipiravir treatment improved clinical pneumonia, high virologic clearance, and safety [13,14,15,16,17]. Moreover, favipiravir treatment eliminated SARS-CoV-2 and reduced hospitalization, including mechanical ventilation [18]. Favipiravir treatment shortened the time to the cessation of viral shedding to 5 days and the time to clinical cure within 3 days [19]. A recent study showed the failed efficacy of favipiravir treatment in mild-to-moderate COVID-19; however, the results of more than 80% of Hispanic and 76% of overweight to obese patients might be contrary to the non-linear pharmacokinetics of favipiravir [20,21]. A meta-analysis showed that favipiravir treatment was safe and provided significant clinical improvement, lower clinical deterioration, greater viral clearance, reduced supplemental oxygen therapy, and lower mortality [22,23]. According to the disruption of healthcare communication technologies during the SARS-CoV-2 pandemic, telehealth provided a critical role in emergency responses; however, there were rare reports about telehealth used in outpatient COVID-19 cases and home-isolated care during the outbreak [24]. The role of favipiravir treatment and using telecare in mild to moderate COVID-19 for preventing clinical deterioration in home isolated patients remained an interesting topic and was the aim of this study.

## 2. Methods

A single-center, retrospective cohort study was conducted from 10 April 2021 to 9 May 2021 at Chulabhorn Hospital, Bangkok, Thailand. The medical records and electronic data were reviewed. Medical consent for treatment was obtained without consent to participate, which was waived by the ethics committee. The study was approved by the Ethics Committee for Human Research, Chulabhorn Research Institute, no. 060/2564. 

### 2.1. Study Subjects

**Figure 1:**
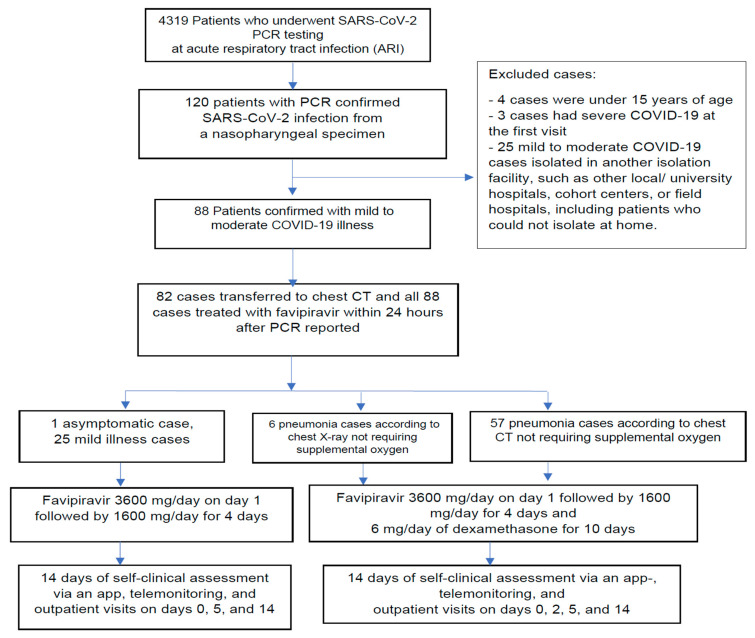
Flow chart of patient selections.

As a part of the standard of care, 4319 patients underwent SARS-CoV-2 polymerase chain reaction (PCR) testing. Eighty-eight PCR-confirmed COVID-19 cases were in accordance with the eligibility criterion of mild to moderate COVID-19. The COVID-19 severity classification was modified from the Infectious Diseases Society of America and National Institute of Health guidelines. Mild illness was defined as symptomatic cases with at least one of the following respiratory symptoms: fever, cough, myalgia, sore throat, runny nose, dyspnea, chest discomfort, and shortness of breath. Moderate illness was defined as all pneumonia cases not receiving supplemental oxygen [25,26] (Figure 1).

This study excluded patients with severe illness on the first visit, patients aged under 15 years, and those who were quarantined in other facilities (Figure 1). Severe COVID-19 illness was defined as pneumonia with a respiratory rate > 30 breaths per minute, oxygen saturation ≤94% at rest, >50% pulmonary infiltrates, requiring supplemental oxygenation, respiratory failure, shock, multi-organ failure, COVID-19 severity index >4, and World Health Organization (WHO) clinical progression scale >3 [6,25,26,27,28].

### 2.2. Measurements

All 88 patients consented to the home isolation. In an exclusively telemonitoring program, patients were required to perform an online self-clinical assessment, and home oxygenation saturation monitoring was recorded via an application. Well-trained nurses performed the daily video call via the app for symptoms and severity assessment, especially in cases with escalated severity or symptom progression from the self-clinical assessment reports. Patients who developed severe illness could access the 24 h emergency medical services (EMS) and be promptly transferred to the hospital. All patients underwent clinical, laboratory, virologic evaluations, and radiologic assessments by the physicians on days 2, 5, and 14 for moderate illness and days 5 and 14 for mild illness at the outpatient COVID-19 clinic.

The cycle threshold (Ct) values of SARS-CoV-2 PCR from a nasopharyngeal (NP) specimen indicated a viral endpoint on days 0, 5, and 14. All confirmed cases were transferred for chest computed tomography (CT), which radiologists interpreted. Chest CT was found to be effective in distinguishing between patients with common types of COVID-19 and those with severe forms of the disease. Chest CT was used in accurately assessing and differentiating the severity of lung lesions in COVID-19 patients, which can aid in the clinical management and treatment decision-making [29]. To prioritize patient safety and facilitate the early detection of the dynamics of deterioration, all patients underwent chest X-rays for comparative analysis on specific follow-up days, including day 0, 2, 5, and 14 of their treatment. This requirement remains in place despite the initial chest CT scan performed during the patient’s first visit. By adhering to this protocol, healthcare providers can proactively monitor patients’ progress and promptly identify any concerning changes or abnormalities that may arise during treatment. The utilization of chest X-rays at designated intervals allows for effective comparisons between different time points, enabling timely interventions and appropriate adjustments to the treatment plan. 

Clinical improvement was defined as the improvement of two or more symptoms >24 h after starting treatment. Clinical deterioration was defined as one or more symptoms that worsened >24 h after starting treatment, and stable disease was defined as less than two symptoms that improved or no change in symptoms.

### 2.3. Treatment

No specific global standard COVID-19 treatment protocol has been established. A COVID-19 treatment protocol was developed in Chulabhorn Hospital as the local standard of care regimen. Patients with mild illness received 3600 milligrams (mg) of favipiravir on the first day, then 1600 mg per day for 4 days. All moderate illness patients received a combination of favipiravir and dexamethasone 6 mg/day for 10 days. Patients with obesity with a body mass index (BMI) > 35 kg/m^2^ received 4800 mg of favipiravir on the first day, followed by 2000 mg per day for 9 days. All patients who were followed up and who developed rapidly progressive pneumonia, tachypnea (>26 breaths/minutes), and radiologic findings indicating >50% pulmonary involvement received 18 mg of dexamethasone per day. 

### 2.4. Statistical Analysis

Continuous, non-normally distributed data were presented as the median (interquartile range (IQR)). Categorical data were analyzed using the Chi-squared test or Fisher’s exact test. Non-normally distributed continuous data were compared using the Mann–Whitney U test or Kruskal–Wallis test for multiple groups. Univariate and multivariate Cox regression models were used to identify the risk factors for clinical deterioration or death, and hazard ratios (HR) and 95% CIs were reported. A two-sided *p* value < 0.05 was considered statistically significant, and all statistical analyses were performed with STATA, version 16.1 (StataCorp, College Station, TX, USA). It was impossible to obtain some data due to the lack of participant follow-up according to local quarantine Thai national policies.

## 3. Results

### 3.1. Baseline Characteristics

This study comprised 17–72-year-old patients. All 42 random patients had the Alpha variant (B.1.1.7). Comorbidities comprised mainly diabetes mellitus, obesity, and hypertension. Eighty-two cases underwent chest CT, revealing pneumonia in 69.51% of the patients, 59.6% with multifocal ground-glass opacities, and 33.3% with multifocal consolidation. Chest X-ray interpretation by radiologists demonstrated pneumonia in 35.23% of the patients. The discordant pneumonia detection rate between chest CT and chest X-ray was 29.55%.

The median high-sensitivity C-reactive protein (hsCRP) was 5.96 mg/L (IQR: 2.1, 17) in the moderate illness group and 1.1 mg/L (IQR: 0.77, 3.16) in the mild illness group.

COVID-19 severity was classified as a moderate illness in 71.59% of the patients, mild illness in 27.27%, and asymptomatic in 1.13%. The median WHO clinical progression scale was 2. In pneumonia cases, 87.3% of the patients had a CURB-65 score of 0, with a score of 1 in 12.7% of patients. The median Ct value from SARS-CoV-2 PCR was 20.85 (IQR: 18.34, 26.12) (Table 1).

### 3.2. Treatment Results

The patients were treated with favipiravir on a median day 4, IQR (2, 7.5), from the onset of symptoms. The 62 patients (71.26%) demonstrated no clinical deterioration within day 5 after favipiravir treatment, comprising 66.67% of moderate illness cases and 88.33% of mild illness cases. 

On days 5 and 14 after treatment, clinical improvement was observed in 50% and 85.51% of the moderate illness patients and 54.17% and 91.66% of the mild illness patients, respectively. A value of ≤2 on the WHO clinical progression scale was recorded for 90.47% and 98.41% of the moderate illness patients and 100% and 100% of the mild illness patients on days 5 and 14, respectively. (Table 2).

Eleven (12.5%) patients required hospitalization and supplemental oxygen; all were moderate COVID-19 cases, with 7.95% receiving an oxygen cannula and 1.14% receiving mechanical ventilation. The intensive care unit (ICU) admission rate was 1.14%, and the 14-day all-cause mortality rate was 1.1%, comprising a patient who died from end-stage renal disease with hyperkalemia. No patients died from severe COVID-19. Pneumonia resolution on day 5 was 43.75% (Table 3).

No difference in clinical deterioration on day 5 was observed in early (<5 days after symptoms onset) and late favipiravir treatment groups. Clinical improvement in the early treatment group occurred in 43.18%, 68.29%, and 86.27% of the patients on days 2, 5, and 14, respectively. 

**Figure 2:**
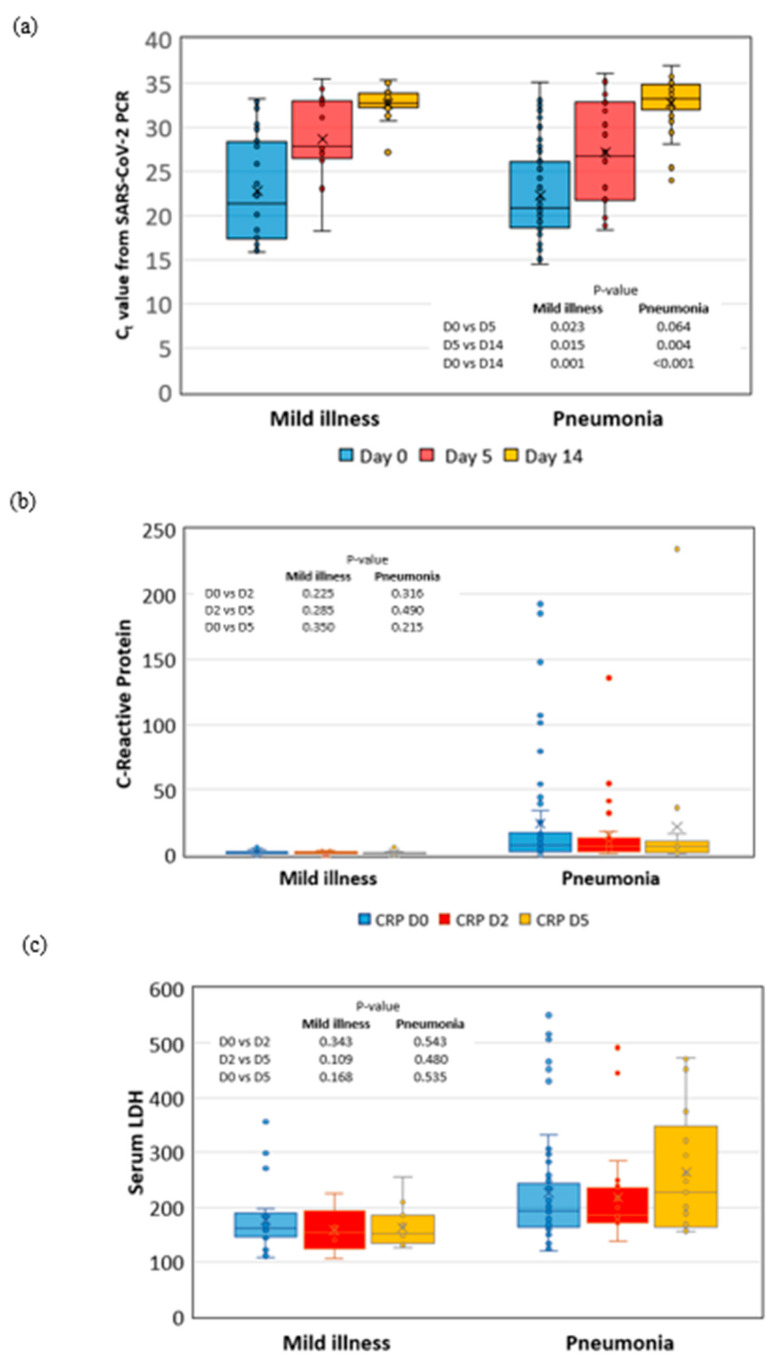
Dynamic assessment of viral shedding represented by (**a**) cycle threshold (Ct) values from SARS-CoV-2 PCR, which showed a significant decrease between day 0, day 5 vs. day 14. The changing of inflammatory markers is included. (**b**) Highly sensitive C-reactive protein (hs-CRP), and (**c**) lactate dehydrogenase (LDH) demonstrated no significant change between day 0, 2, and 5 after favipiravir treatment.

The Ct values on days 0, 5, and 14 did not differ in mild and moderate COVID-19 patients. The median Ct values showed a statistically significant virologic response in both mild illness and pneumonia between day 0 vs. day 14 (*p* = 0.001, *p* < 0.001) and day 5 vs. day 14 (*p* = 0.015, *p* = 0.004). However, the only significant change in Ct value in mild illness patients between day 0 and 5. (*p* = 0.023) (Figure 2). 

Moreover, the virologic clearance in mild illness was not different from the moderate illness on day 14 (*p* = 1.000) (Table 2).

Multivariate analysis showed that the Ct values from SARS-CoV-2 PCR were associated with pneumonia (*p* < 0.001). Univariate analysis revealed that BMI, hsCRP, lactate dehydrogenase, ferritin, and Ct values from SARS-CoV-2 PCR were associated with pneumonia, with odds ratios of 1.12, 1.34, 1.01, 1.00, and 294 (*p* = 0.034, 0.009, 0.046, 0.02, <0.001), respectively. Univariate analysis demonstrated that clinical deterioration was not associated with the elderly (*p* = 0.253), diabetes (*p* = 0.634), or obesity (*p* = 0.489) (Table 4).

In contrast, a subgroup analysis in clinically deteriorated patients was associated with dyspnea (*p* = 0.023; 95% CI [1.19–11.77]) and chest discomfort (*p* = 0.031; 95% CI [1.11–9.43]). Elevated initial hsCRP concentrations (odds ratio (OR): 1.01; *p* = 0.004; 95% CI: [1.00–1.02]) and low total lymphocyte count (OR: 1.00; *p* = 0.004; 95% CI: 1.00–1.00) tended to be associated with poor clinical outcomes. There was a higher proportion of patients without clinical deterioration who initiated favipiravir after >3 days (59.74%) than those who initiated favipiravir after >5 days (40.26%) of onset of illness (Table 5).

Adverse events comprised nausea in 3 (3.4%) cases and hepatitis in 11 (12.5%) cases; mild hepatitis occurred in 6 (6.8%) cases, and moderate hepatitis occurred in 5 (5.7%) cases, which were all resolved without sequelae.

## 4. Discussion

This study evaluated the outcomes of favipiravir treatment in mild to moderate COVID-19 patients undergoing home isolation and telemonitoring. On day 5 of favipiravir treatment, 71.26% of the cases demonstrated no clinical deterioration, namely 66.67% and 88.33% of the moderate and mild COVID-19 patients. Although our study included a relatively small sample size of only 42 randomly selected respiratory tract samples, it is important to highlight that the study period coincided with the prevailing dominance of the Alpha variant [30]. In a large population study, favipiravir treatment prevented deterioration to severe disease in 79.1% of the patients, 76.9% of moderate cases, and 86.2% of mild cases on day 7 [17]. A previous report showed a rate of 16.8% disease deterioration to severe illness during hospitalization; however, 25% of the cases received a three-drug regimen (favipiravir, lopinavir/ritonavir, or darunavir/ritonavir, with hydroxychloroquine) [31,32]. A randomized trial showed the failed efficacy of favipiravir in non-severe COVID-19 treatment; however, the study consisted of 80% of White and Hispanic patients and 76% of patients with a BMI > 25 kg/m^2^ might be concerned about inadequate dose from the non-adjusted dose of favipiravir per body weight based on the non-linear pharmacokinetics of favipiravir knowledge [20,21].

Obesity, advanced age, and D-dimers associated with COVID-19 pneumonia described in the recent study were not linked to clinical deterioration in our study [30]. Our study revealed that BMI, hs-CRP, LDH, ferritin, and Ct values were related to developing pneumonia, which correlated with a recent study [33].

Clinical improvement in moderate and mild illness on day 5 occurred in 50% and 54.17% of the patients. The overall clinical improvement in the early favipiravir group occurred in 68.29% of the patients on day 5 of treatment, which was an interesting outcome compared with values of 67.7% and 71.4% on day 7 in previous studies, although the assessment days varied between the studies [14,28]. 

Telehealth and self-clinical assessment could identify more symptomatic cases. Less than 10% of patients were reported as asymptomatic in a previous study [31]. In our study, no mild cases developed pneumonia, required supplemental oxygen, or reported an escalated WHO clinical progression grade.

Pneumonia was detected in 71.5% of cases at the first visit, on average 4 days after the onset of symptoms, which was a higher rate of early pneumonia detection than in other reports [31,34,35,36,37]. The radiologic discordance between plain X-rays and chest CT was very high. Our study indicated that general practices might under-evaluate COVID-19 pneumonia from chest radiography, leading to unfavorable outcomes. Dedicated chest CT helped triage patients and decide on treatment when available. Notably, the pneumonia resolution rate on day 5 in this study was higher than in previous studies [17]. In resource-limited situations, clinical characteristics and hsCRP in negative chest radiograph patients might be used instead of chest CT to determine pneumonia and severity assessment to initiate treatment. 

This study demonstrated that supplemental oxygen was required in 12.5% of moderate COVID-19 cases; only one case developed acute respiratory distress syndrome (ARDS), and the patient survived. A recent study revealed that 18.1% of pneumonia cases detected through chest CT necessitated supplemental oxygen. However, an intriguing finding in that study was that among the patients receiving favipiravir treatment during the COVID-19 outbreak, only 46.55% were confirmed as COVID-19 cases, despite being treated during the outbreak [14]. Moreover, our study showed a lower supplemental oxygen rate than a previous study that reported that 27% of the patients required an oxygen cannula, and 6.4% required mechanical ventilation [28].

The ICU admission rate was low in our study compared with 7–10.8% in a recent study of patients treated with favipiravir [38], of which 18.2% received favipiravir with interferon beta-1b by inhalation aerosol, and 17.8% received favipiravir and hydroxychloroquine [39]. Our study revealed that the 14-day mortality rate from severe pneumonia was 0%, although the all-cause mortality was 1.1% from not receiving dialysis in an end-stage renal disease patient. In contrast, a recent study indicated a 13.2% mortality rate in moderate and 3.6% in mild COVID-19 patients [17]. However, another study in which patients were treated with favipiravir combined with another antiviral agent indicated a 12.5% mortality rate [28].

Our study did not show a difference between early and late favipiravir treatment. Although, previous data showed that early favipiravir treatment (<4 days after symptom onset) showed a positive correlation between the duration of fever defervescence and early time to initiate favipiravir treatment [40]. However, the early-initiated favipiravir treatment after ≥3 days demonstrated less clinical deterioration than treatment initiated after ≥5 days, implying a favorable outcome in the earlier treatment group.

The open-label randomized controlled trial (RCT) conducted in Malaysia revealed that the use of favipiravir did not effectively prevent the progression of COVID-19 to a severe form of the disease. The study reported that 18% of patients receiving favipiravir developed severe disease, compared to 14.8% in the control group. It is important to note that both groups initiated favipiravir treatment five days after the onset of illness, and at baseline, 16.4% of the participants required supplemental oxygen. Furthermore, the study reported several instances of contamination with other antiviral or immunomodulating agents, which potentially confounded the results. Additionally, the study observed that 20% of patients in both groups were obese [41]. An additional small-scale RCT yielded negative results regarding the clinical efficacy of favipiravir in treating symptomatic mild to moderate cases of COVID-19. However, it is important to highlight that this study suffered from insufficiently detailed descriptions of key factors, including baseline characteristics, such as comorbidities, pulmonary involvement, vaccination status, and symptoms experienced in the five days leading up to the study. It is worth mentioning that the majority of patients included in this study had a WHO clinical progression scale score of 2 [42].

Recent RCTs have presented conflicting findings regarding the efficacy of favipiravir. One study focused on mild COVID-19 patients and utilized a fixed-dose regimen of 1800 mg of favipiravir twice daily on the first day, followed by a maintenance dose of 800 mg twice daily for 5 to 7 days, regardless of body weight. The results of this study demonstrated no significant differences in terms of viral clearance time, time to recovery, hospitalization rates, and ICU admissions between the favipiravir-treated group and the control group. However, it is worth noting that within the favipiravir-treated group, 21.4% of patients had a body mass index (BMI) greater than 30 kg/m^2^, without any adjustment in the dosage of favipiravir [43]. In contrast, our study observed a lower percentage of obese patients, specifically 13.6%.

The PRESECO study failed to demonstrate the benefits of early favipiravir treatment in mild-to-moderate COVID-19 cases, as evidenced by the time to sustained clinical recovery in various demographic groups, including 80% of Hispanic and White patients and a quarter of seropositive individuals. Additionally, the study reported that 76% of overweight patients (BMI > 25 kg/m^2^) were included. However, due to the complex non-linear pharmacokinetic profile of favipiravir, it is possible that the dosage used in the study was relatively low, particularly for obese patients, resulting in suboptimal therapeutic concentrations [20]. In a recent study involving COVID-19 cases confirmed by SARS-CoV-2 PCR testing, in 46.5% of patients, it was found that 18.1% of individuals treated with favipiravir required supplemental oxygen. Chest CT scans detected pneumonia in the favipiravir-treated group [14]. The hospitalization rate for mild COVID-19 cases treated with favipiravir was reportedly 5.3% [43]. Another study focused on high-risk mild to moderate COVID-19 cases revealed that 18.4% of patients in the favipiravir-treated group required supplemental oxygen, and 2.4% required mechanical ventilation [41]. Furthermore, a meta-analysis showed that using favipiravir reduced the need for supplemental oxygen by 7% compared to the control group, 4% of patients in the favipiravir-treated group [22]. In our study, a lower proportion of mild to moderate COVID-19 patients required hospitalization and supplemental oxygen, which may be attributed to the early initiation of loading and maintenance dose-adjusted favipiravir treatment.

Despite well-designed studies, the treatment outcomes of favipiravir have shown interpersonal variability. Pharmacokinetic (PK) data indicate that the trough concentration of favipiravir substantially decreases over time in patients with COVID-19, particularly those with a BMI > 25 kg/m^2^. In a Turkish study, the trough concentration decreased by 89% between day 2 (21.26 μg/mL) and day 4 (1.6 μg/mL). It is important to note that a trough concentration greater than 20 μg/mL is necessary to achieve the 50% effective concentration (EC50) value of 9.4 μg/mL. Conversely, in healthy volunteers receiving a standard dose regimen of 600 mg twice daily on day 1, followed by 600 mg twice daily on days 2–5, the trough concentration ranged from 20 to 60 μg/mL [44,45,46,47]. A population PK study demonstrated that factors such as body surface area, dosage, and invasive mechanical ventilation influence the clearance and bioavailability of favipiravir. The study also indicated that a double dose regimen of 3200 mg/1200 mg twice daily resulted in a lower trough concentration than the EC50 value. However, in a patient with a body surface area of 1.72 but not a BSA value equal to 2.2, a regimen of 1600 mg twice daily exceeded the EC50 value. These findings suggest that a maintenance dose of favipiravir between 800 and 1200 mg twice daily may be sufficient to achieve therapeutic concentrations of the active intracellular favipiravir metabolite [47].

Our study suggests that the potential clinical benefits of favipiravir treatment can be realized under specific conditions, including appropriate loading and maintenance dosages adjusted to body weight and early initiation within 72 h of illness onset. Additionally, our findings indicate that Asian ethnicity may be a significant factor in predicting favorable outcomes of favipiravir treatment for mild-to-moderate COVID-19 cases compared to Americans and Caucasians. It is worth noting that the 50% plasma concentration of favipiravir observed in Japanese individuals served as a benchmark [48]. Favipiravir can be considered as a treatment option for individuals who have significant drug interactions with nirmatrelvir/ritonavir, express concerns about mutagenesis in mammalian cells, or reside in resource-limited countries that face outbreaks of newly emerging variants of concern.

In contrast to recent studies, neither obesity nor diabetes mellitus were significant predictive factors for deterioration in our study [6]. The RECOVERY trial data supported the benefit of corticosteroid treatment in hospitalized patients, especially those who required supplemental oxygen. Our data on the potential benefit of early favipiravir or steroids in outpatient pneumonia patients must be debated [49].

Prolonged viral shedding in patients receiving corticosteroids is a concern, although the benefit of corticosteroids is reduced mortality and mechanical ventilation needed. Some studies extended favipiravir treatment to 10 days in the pneumonia treatment regimen to reduce viral shedding [49,50]. In our study, we administered a 10-day course of favipiravir and dexamethasone to all patients with pneumonia. The extended use of favipiravir aimed to mitigate prolonged viral shedding, while the inclusion of corticosteroids was intended to harness their anti-inflammatory properties and prevent cytokine storms in pneumonia patients. This approach ensured that the home isolation program was safe and effective in preventing severe deterioration.

However, our study showed no difference in viral clearance on days 5 and 14 in mild and moderate COVID-19 patients. The Ct values from SARS-CoV-2 PCR implied that favipiravir might achieve rapid virologic clearance in moderate and mild illness groups on days 5 and 14. Udwadia’s study showed cessation of oral shedding at rates of 97.7% and 96.4% in mild and moderate COVID-19 patients, respectively, which is similar to another study showing a 62.5% rapid viral clearance rate within 4 days; however, the initial low viral burden Ct value was 30.7 (IQR: 28.0–33.3) in the favipiravir arm [13,15,19]. Our findings were consistent with those reported in the work of Doi et al., in which favipiravir did not significantly demonstrate viral clearance on day 5 [51]. In our study, the cessation rate of NP SARS-CoV-2 shedding was 36.2% in pneumonia patients on day 14. The clinical implication is that COVID-19 patients who receive prolonged high-dose steroid treatment might prolong antiviral therapy to ≥10 days for adjuvant activity in achieving virologic clearance. 

Under social distancing situations and avoiding cross-contamination, telemonitoring and telecare adjunct to outpatient antiviral treatment demonstrated benefits in preventing the deterioration of COVID-19 severity. The essential factors were well-educated healthcare professionals who use telehealth, providing dedicated healthcare systems including emergency medical services, inpatient and intensive care transfer, COVID-19 outpatient departments, home monitoring programs, patient-generated health data, real-time data collection and visualization, mobile health applications and the selection of suitable individuals [8,24,52].

Notably, the limitations of this study are the small number of cases and retrospective data. Only 9.1% of the elderly patients and all the Alpha variants patients were presented. Corticosteroids were administered to all pneumonia patients. Although corticosteroids’ role is controversial, randomized controlled trials are needed to prove the benefits of adjunctive treatment in outpatient pneumonia cases. We extended the duration of favipiravir treatment because of concern regarding prolonged viral shedding.

## 5. Conclusions

The administration of favipiravir for the treatment of mild to moderate cases of COVID-19 demonstrated both safety and efficacy in preventing clinical deterioration, including the need for supplemental oxygen when adjunct with telemonitoring. Therefore, favipiravir might be a potential anti-SARS-CoV-2 agent to mitigate the progression of the disease and alleviate the burden on healthcare systems [53].

## Figures and Tables

**Table 1:** Patients’ baseline characteristics.

Characteristics	N = 88 (%)
Male gender, n (%)	43 (48.86)
Age, years, median (IQR)	32 (26, 52)
Age > 60, years, n (%)	8 (9.1)
Alpha variant (B.1.1.7), n/total (%)	42/42 (100)
Comorbidity, n (%)	31(35.2)
Diabetes mellitus	12 (13.64)
Obesity	12 (13.64)
Hypertension	11 (12.50)
Asthma	4 (4.5)
Chronic lung disease	1 (1.14)
End-stage renal disease	1 (1.14)
Other	5 (5.68)
BMI, n (%)	
<18.5 kg m^−2^	9 (10.98)
18.5–24.9 kg m^−2^	31 (37.80)
25–29.9 kg m^−2^	30 (36.59)
30–34.9 kg m^−2^	8 (9.76)
≥35 kg m^−2^	4 (4.88)
Resting oxygen saturation on day 0 (%), median (IQR)	98 (97, 99)
Respiratory rate (per minute), median (IQR)	20 (20, 20)
Heart rate (bpm), median (IQR)	90 (79, 100)
Symptom at baseline, n (%)	
Cough	50 (56.82)
Dyspnea	28 (31.82)
Chest pain/Chest discomfort	18 (20.45)
Fever	34 (38.64)
Loss of appetite	62 (71.26)
Sore throat	38 (43.18)
Runny nose	31 (35.23)
Myalgia	34 (38.64)
Diarrhea	21 (24.42)
Anosmia or ageusia	17 (19.54)
Skin rash	4 (4.60)
Conjunctivitis	1 (1.16)
Chest X-ray indicating pneumonia, interpreted by a radiologist, n (%)	31 (35.23)
Chest CT indicating pneumonia, n (%)	57 (64.77)
Several ground-glass opacities in both lungs	34/57 (59.6)
Multiple consolidations in both lungs	19/57 (33.33)
Focal ground-glass opacities	9/57 (15.79)
Focal consolidations	4/57 (7.02)
Discordance between chest CT and chest X-ray, n (%)	26 (29.55)
SARS-CoV-2 PCR Ct value, median (IQR)	20.85 (18.34, 26.12)
SARS-CoV-2 PCR Ct value mild illness/pneumonia, median (IQR)	20.79 (18.3, 26.6)/20.9 (18.3, 26.7)
Total WBC count (×10^9^ cells/L), median (IQR)	5.100 (4240, 5950)
Total lymphocyte count (cells/mm^3^), median (IQR)	1556 (1208, 1936)
Mild illness	1669.34 (1288.74, 1826.5)
Moderate illness	1548.72 (1196.8, 2012.64)
hsCRP at baseline (mg/L), median (IQR)	3.63 (1.25, 13.29)
Mild illness	1.1 (0.77, 3.16)
Moderate illness (pneumonia)	5.96 (2.1, 17)
Characteristics	N = 88 (%)
Ferritin (ng/mL), median (IQR)	
Mild illness	136 (92.4, 282)
Moderate illness	344.75 (137.85, 606.1)
COVID-19 severity, n (%)	
-Asymptomatic	1 (1.13)
-Mild illness	24 (27.27)
-Moderate illness (pneumonia without requiring oxygen)	
WHO COVID-19 severity score, median (IQR)	2 (2, 2)
COVID-19 severity index, median (IQR)	2 (1, 2)
CURB-65 score, n (%)	
CURB 65 = 0	55 (87.3)
CURB 65 = 1	8 (12.7)
The median number of days from symptoms to starting favipiravir (IQR)	4 (2, 7.5)
Duration of favipiravir after symptom onset: ≤5 days/>5 days, n (%)	53 (60.23)/35 (39.77)
SARS-CoV-2 IgG seroprevalence, n/total n (%)	9/78 (11.54)

Abbreviation: N: number of SARS-CoV-2 infected patients; OR: odds ratio; CI: confident interval; Ct value: cycle threshold value; SARS-CoV-2: severe acute respiratory syndrome coronavirus-2; PCR: polymerase chain reaction; BMI: body mass index; IQR: interquartile range; bpm: beat per minute; CT: computed tomography; hsCRP: high-sensitivity C-reactive protein; Ig: immunoglobulin; COVID-19: coronavirus disease 2019; WBC: white blood cells.

**Table 2:** Rates of clinical deterioration, changing COVID-19 severity index, resolution of pneumonia and virological endpoint between patients with mild vs. moderate illness.

Clinical Outcome	Total (N = 87)	Moderate Illness (N = 63)	Mild Illness (N = 24)	*p*-Value
No clinical deterioration on day 5 of favipiravir therapy, n (%)	62 (71.26)	42 (66.67)	20 (83.33)	0.125
Clinical improvement within 5 days of treatment, n (%)	45 (51.72)	32 (50.79)	13 (54.17)	0.778
Clinical improvement within 14 days of treatment, n (%)	76 (87.36)	54 (85.71)	22 (91.66)	0.720
Requiring oxygen after day 2 of treatment, n (%)	9 (10.34)	9 (14.29)	0	0.058
Requiring oxygen after day 5 of treatment, n (%)	2 (2.29)	2 (3.17)	0	1.000
WHO clinical progression scale ≤2 on day of initial treatment, n (%)	81 (93.10)	57 (90.47)	24 (100)	0.181
WHO clinical progression scale ≤2 on day 14, n (%)	86 (98.85)	62 (98.41)	24 (100)	1.000
COVID-19 severity index did not deteriorate after:				
48 h, n (%)	72 (82.75)	51 (80.95)	21 (87.5)	0.545
5 days, n (%)	78 (89.66)	55 (87.30)	23 (95.83)	0.434
14 days, n (%)	82 (94.25)	58 (92.06)	24 (100)	0.555
Resolution of pneumonia according to chest CT on day 5, n (%) (n = 16)	7 (43.75)	7(43.75)	0 (0)	-
SARS-CoV-2 PCR undetected on day 14, n. (%)	28/76 (36.8)	20/61 (32.7)	8/25 (32)	1.000
SARS-CoV-2 PCR Ct value on day 0, median (IQR)	20.8 (18.3, 26.7)	20.9 (18.3, 26.7)	20.8 (18.3, 26.6)	0.371
SARS-CoV-2 PCR Ct value on day 5, median (IQR)	27.6 (23.1, 32.8)	27.4 (23.1, 32.9)	27.4 (23.1, 32.8)	0.223
SARS-CoV-2 PCR Ct value on day 14, median (IQR	32.8 (32.1, 34.4)	32.8 (32.1, 34.3)	32.8 (32.1, 34.3)	0.439

Abbreviation: N: the total number of SARS-CoV-2 infected patients; n: number of patients; CT: computed tomography; WHO: World Health Organization; Ct value: cycle threshold value; COVID-19: coronavirus disease 2019; SARS-CoV-2: severe acute respiratory syndrome coronavirus-2; PCR: polymerase chain reaction; IQR: interquartile range.

**Table 3:** Clinical endpoints of clinically deteriorated patients between the mild and moderate illness (pneumonia) groups.

Clinical Endpoints	Total (N = 87) n (%)	COVID-19 Severity		*p*-Value
		Mild Illness (N = 24) n (%)	Pneumonia (N = 63) n (%)	
ICU admission within 14 days	1 (1.14)	0 (0.00)	1 (1.59)	1.000
Hospitalization	11 (12.5)	0 (0.00)	11 (100.00)	0.030
Oxygen therapy	11 (12.50)	0 (0.00)	11 (100.00)	0.030
-Oxygen cannula	7 (7.95)	0 (0.00)	7 (11.11)	0.184
-HFNC	2 (2.27)	0 (0.00)	2 (3.17)	1.000
-NIPPV	1 (1.14)	0 (0.00)	1 (1.59)	1.000
-Mechanical ventilation	1 (1.14)	0 (0.00)	1 (1.59)	1.000
14-day all-cause mortality	1 (1.14 *)	0 (0.00)	1 (1.59)	1.000
14-day mortality from severe COVID-19 pneumonia	0 (0)	0 (0.00)	0 (0.00)	1.000

* Death from end-stage renal disease without dialysis, with electrolyte imbalance. Abbreviation: N: the total number of SARS-CoV-2 infected patients; n: number of patients; NIPPV: non-invasive positive-pressure ventilation; COVID-19: coronavirus disease 2019; ICU: intensive care unit; HFNC: high-flow nasal cannula.

**Table 4:** Univariate and multivariate analysis of the factors associated with pneumonia.

Parameter	Univariate Analysis		Multivariate Analysis	
	Odds Ratio (95% CI)	*p*-Value	Odds Ratio (95% CI)	*p*-Value
BMI	1.12 (1.0089–1.2525)	0.034	0.86 (0.6699–1.1110)	0.253
hsCRP	1.34 (1.0760–1.6708)	0.009	0.98 (0.8649–1.1243)	0.834
Ferritin	1.00 (1.0004–1.0053)	0.021	1.00 (0.9983–1.0100)	0.170
LDH	1.01 (1.0002–1.0215)	0.046	1.00 (0.9831–1.0213)	0.835
Ct value from SARS-CoV-2 PCR	294 (31.0505–2783.724)	<0.001	513.75 (18.3511–14382.81)	<0.001

Abbreviation: OR: odds ratio, CI: confident interval; BMI: body mass index; hsCRP: high-sensitivity C-reactive protein; LDH: lactate dehydrogenase; Ct value: cycle threshold value; SARS-CoV-2: severe acute respiratory syndrome coronavirus-2; PCR: polymerase chain reaction.

**Table 5:** Subgroup analysis of the factors related to clinical deterioration in patients with mild to moderate COVID-19.

Factors Related to Clinical Deterioration	N (%)	Clinical Deterioration	No Clinical Deterioration	OR (95% CI)	*p*-Value
Male gender, n (%)	43 (48.86)	7 (63.64)	36 (46.75)	1.83 (0.58–5.81)	0.305
Age ≥60 years	8 (9.20)	2 (18.18)	6 (7.89)	2.19 (0.57–8.54)	0.253
Comorbidity, n (%)					
Diabetes mellitus	12 (13.64)	2 (18.18)	10 (12.99)	1.41 (0.34, 5.74)	0.634
Hypertension	11 (12.50)	3 (27.27)	8 (10.39)	2.63 (0.82, 8.43)	0.105
Obesity (BMI ≥ 30 kg/m^2^)	12 (14.63)	2 (22.22)	10 (13.70)	1.67 (0.39, 7.09)	0.489
Duration of COVID-19 symptoms					
≥5 days; n (%)	26 (29.55)	4 (36.36)	22 (28.57)	1.36 (0.44–4.26)	0.595
≥7 days; n (%)	14 (15.91)	2 (18.18)	12 (15.58)	1.17 (0.28–4.87)	0.824
Respiratory rate ≥ 22 min^−1^	68 (80.00)	8 (72.73)	60 (81.08)	0.67 (0.20–2.25)	0.513
Heart rate ≥ 100 bpm	23 (27.71)	4 (36.36)	19 (26.39)	1.49 (0.48–4.62)	0.439
Most severe COVID-19 symptom at baseline, no. (%)					
Cough	50 (56.82)	9 (81.82)	41 (53.52)	3.42 (0.78–14.92)	0.102
Dyspnea	28 (31.82)	7 (63.64)	21 (27.27)	3.75 (1.19–11.77)	0.023
Chest discomfort	18 (20.45)	5 (45.45)	13 (16.88)	3.24 (1.11–9.43)	0.031
Fever	34 (38.64)	7 (63.64)	27 (35.06)	2.78 (0.88–8.79)	0.082
Pneumonia, n (%)	63 (71.59)	20 (83.33)	43 (67.19)	1.98 (0.75–5.23)	0.166
WHO COVID-19 severity score, median (IQR)	2 (2, 2)	2 (2, 2)	2 (2, 2)	-	-
COVID-19 severity index, median (IQR)	2 (1, 2)	2 (2, 5)	2 (1, 2)	-	-
Ct value for COVID-19, median (IQR)	20.85 (18.34, 26.12)	21.80 (18.55, 27.26)	20.72 (18.23, 25.58)	1.02 (0.92–1.13)	0.688
hsCRP at baseline (mg/L), median (IQR)	3.63 (1.25, 13.29)	34.19 (4.22, 54.45)	3.36 (1.09, 10.42)	1.01 (1.00–1.02)	0.004
Ferritin (ng/ml), median (IQR)	282 (113.2, 546.2)	333.95 (189, 557.7)	255.8 (101.2,546.2)	1.00 (1.00–1.00)	0.592
Total lymphocyte count (cells/mm^3^), median (IQR)	1560.2 (1239.2, 1961.7)	1239.52 (799.4, 1461.2)	1669.34 (1269.3, 2018.4)	1.00 (1.00–1.00)	0.004
Time from symptom onset to favipiravir, days, median (IQR)	4 (2, 7.5)	4 (3, 8)	4 (2, 7)	1.00 (0.85–1.17)	0.953
Favipiravir initiation from symptom onset (early vs. late)					
≥3 days, n (%)	54 (61.36)	8 (72.73)	46 (59.74)	1.68 (0.48–5.00)	0.419
≥5 days, n (%)	35 (39.77)	4 (36.36)	31 (40.26)	0.87 (0.27–2.74)	0.184

Abbreviation: N: number of SARS-CoV-2 infected patients; OR: odds ratio; CI: confident interval; IQR: interquartile range; Ct value: cycle threshold value; hsCRP: high-sensitivity C-reactive protein; WHO: World Health Organization; COVID-19: coronavirus disease 2019; SARS-CoV-2: severe acute respiratory syndrome coronavirus-2; BMI: body mass index; bpm beat per minute

## Data Availability

Data sharing not applicable. No new data were created or analyzed in this study. Data sharing si not applicable to this article.

## References

[1] Zhu N., Zhang D., Wang W., Li X., Yang B., Song J., Zhao X., Huang B., Shi W., Lu R. (2020). A novel coronavirus from patients with pneumonia in China, 2019. N. Engl. J. Med..

[2] Wu Z., McGoogan J.M. (2020). Characteristics of and important lessons from the coronavirus disease 2019 (COVID-19) outbreak in China: Summary of a report of 72,314 cases from the Chinese Center for Disease Control and Prevention. JAMA.

[3] World Health Organization (2022). WHO Coronavirus (COVID-19) Dashboard. https://covid19.who.int/.

[4] Spinner C.D., Gottlieb R.L., Criner G.J., López J.R.A., Cattelan A.M., Viladomiu A.S., Ogbuagu O., Malhotra P., Mullane K.M., Castagna A. (2020). Effect of remdesivir vs. standard care on clinical status at 11 days in patients with moderate COVID-19: A randomized clinical trial. JAMA.

[5] Beigel J., Tomashek K., Dodd L. (2020). Remdesivir for the Treatment of COVID-19—Final Report. N. Engl. J. Med..

[6] Gandhi R.T., Lynch J.B., Del Rio C. (2020). Mild or moderate COVID-19. N. Engl. J. Med..

[7] Zhou S., Hill C.S., Sarkar S., Tse L.V., Woodburn B.M., Schinazi R.F., Sheahan T.P., Baric R.S., Heise M.T., Swanstrom R. (2021). β-d-N4-hydroxycytidine Inhibits SARS-CoV-2 through Lethal Mutagenesis but Is also Mutagenic to Mammalian Cells. J. Infect. Dis..

[8] Vidal-Alaball J., Acosta-Roja R., Hernández N.P., Luque U.S., Morrison D., Pérez S.N., Perez-Llano J., Vèrges A.S., Seguí F.L. (2020). Telemedicine in the face of the COVID-19 pandemic. Aten. Primaria.

[9] Elhennawy A., Alsalem F.A., Bahri S., Alarfaj N. (2021). Telemedicine versus Physical Examination in Patients’ Assessment during COVID-19 Pandemic: The Dubai Experience. Dubai Med. J..

[10] Du Y.X., Chen X.P. (2020). Favipiravir: Pharmacokinetics and concerns about clinical trials for 2019-nCoV infection. Clin. Pharmacol. Ther..

[11] Furuta Y., Komeno T., Nakamura T. (2017). Favipiravir (T-705), a broad spectrum inhibitor of viral RNA polymerase. Proc. Jpn. Acad. Ser. B.

[12] Driouich J.S., Cochin M., Lingas G., Moureau G., Touret F., Petit P.R., Piorkowski G., Barthélémy K., Laprie C., Coutard B. (2021). Favipiravir antiviral efficacy against SARS-CoV-2 in a hamster model. Nat. Commun..

[13] Cai Q., Yang M., Liu D., Chen J., Shu D., Xia J., Liao X., Gu Y., Cai Q., Yang Y. (2020). Experimental treatment with favipiravir for COVID-19: An open-label control study. Engineering.

[14] Chen C., Huang J., Yin P., Zhang Y., Cheng Z., Wu J., Chen S., Zhang Y., Chen B., Lu M. (2020). Favipiravir versus arbidol for COVID-19: A randomized clinical trial. medRxiv.

[15] Ivashchenko A.A., Dmitriev K.A., Vostokova N.V., Azarova V.N., Blinow A.A., Egorova A.N., Gordeev I.G., Ilin A.P., Karapetian R.N., Kravchenko D.V. (2020). AVIFAVIR for Treatment of Patients with Moderate Coronavirus Disease 2019 (COVID-19): Interim Results of a Phase II/III Multicenter Randomized Clinical Trial. Clin. Infect. Dis..

[16] Joshi S., Parkar J., Ansari A., Vora A., Talwar D., Tiwaskar M., Patil S., Barkate H. (2020). Role of favipiravir in the treatment of COVID-19. Int. J. Infect. Dis..

[17] Doi Y.K., Ando M., Kuwatsuka M., Ishihara Y., Favipiravir T. (2021). Favipiravir Observational Study Interim Report 3.

[18] Dabbous H.M., Abd-Elsalam S., El-Sayed M.H., Sherief A.F., Ebeid F.F., El Ghafar M.S.A., Soliman S., Elbahnasawy M., Badawi R., Tageldin M.A. (2021). Efficacy of favipiravir in COVID-19 treatment: A multi-center randomized study. Arch. Virol..

[19] Udwadia Z.F., Singh P., Barkate H., Patil S., Rangwala S., Pendse A., Kadam J., Wu W., Caracta C.F., Tandon M. (2021). Efficacy and safety of favipiravir, an oral RNA-dependent RNA polymerase inhibitor, in mild-to-moderate COVID-19: A randomized, comparative, open-label, multicenter, phase 3 clinical trial. Int. J. Infect. Dis..

[20] Golan Y., Campos J.A.S., Woolson R., Cilla D., Hanabergh R., Gonzales-Rojas Y., Lopez R., Finberg R., Balboni A. (2022). Favipiravir in patients with early mild-to-moderate COVID-19: A randomized controlled trial. Clin. Infect. Dis..

[21] Irie K., Nakagawa A., Fujita H., Tamura R., Eto M., Ikesue H., Muroi N., Fukushima S., Tomii K., Hashida T. (2021). Population pharmacokinetics of favipiravir in patients with COVID-19. CPT Pharmacomet. Syst Pharm..

[22] Hassanipour S., Arab-Zozani M., Amani B., Heidarzad F., Fathalipour M., Martinez-de-Hoyo R. (2021). The efficacy and safety of Favipiravir in treatment of COVID-19: A systematic review and meta-analysis of clinical trials. Sci. Rep..

[23] Shrestha D.B., Budhathoki P., Khadka S., Shah P.B., Pokharel N., Rashmi P. (2020). Favipiravir versus other antiviral or standard of care for COVID-19 treatment: A rapid systematic review and meta-analysis. Virol. J..

[24] Smith A.C., Thomas E., Snoswell C.L., Haydon H., Mehrotra A., Clemensen J., Caffery L.J. (2020). Telehealth for global emergencies: Implications for coronavirus disease 2019 (COVID-19). J. Telemed. Telecare.

[25] Bhimraj A., Morgan R.L., Shumaker A.H., Lavergne V., Baden L., Cheng V.C.C., Edwards K.M., Gandhi R., Muller W.J., O’Horo J.C. (2020). Infectious Diseases Society of America Guidelines on the Treatment and Management of Patients with COVID-19. Clin. Infect. Dis..

[26] COVID-19 Treatment Guidelines Panel Coronavirus Disease 2019 (COVID-19) Treatment Guidelines. National Institutes of Health. https://www.covid19treatmentguidelines.nih.gov/.

[27] Marshall J.C., Murthy S., Diaz J., Adhikari N.K., Angus D.C., Arabi Y.M., Baillie K., Bauer M., Berry S., Blackwood B. (2020). A minimal common outcome measure set for COVID-19 clinical research. Lancet Infect. Dis..

[28] Rattanaumpawan P., Jirajariyavej S., Lerdlamyong K., Palavutitotai N., Saiyarin J. (2020). Real-world experience with favipiravir for treatment of COVID-19 in Thailand: Results from a multi-center observational study. medRxiv.

[29] Guan X., Yao L., Tan Y., Shen Z., Zheng H., Zhou H., Gao Y., Li Y., Ji W., Zhang H. (2021). Quantitative and semi-quantitative CT assessments of lung lesion burden in COVID-19 pneumonia. Sci. Rep..

[30] Muadchimkaew M., Siripongboonsitti T., Wongpatcharawarakul S., Boonsankaew C., Tawinprai K., Soonklang K., Mahanonda N. (2022). Effect of Inactivated SARS-CoV-2 Vaccines and ChAdOx1 nCoV-19 Vaccination to Prevent COVID-19 in Thai Households (VacPrevent trial). Int. J. Infect. Dis..

[31] Sirijatuphat R., Suputtamongkol Y., Angkasekwinai N., Horthongkham N., Chayakulkeeree M., Rattanaumpawan P., Koomanachai P., Assanasen S., Rongrungruang Y., Chierakul N. (2021). Epidemiology, clinical characteristics, and treatment outcomes of patients with COVID-19 at Thailand’s university-based referral hospital. BMC Infect. Dis..

[32] Pongpirul W.A., Wiboonchutikul S., Charoenpong L., Panitantum N., Vachiraphan A., Uttayamakul S., Pongpirul K., Manosuthi W., Prasithsirikul W. (2020). Clinical course and potential predictive factors for pneumonia of adult patients with Coronavirus Disease 2019 (COVID-19): A retrospective observational analysis of 193 confirmed cases in Thailand. PLoS Negl. Trop. Dis..

[33] Özdemir Y.E., Balkan I.I., Bayramlar O.F., Alkan S., Murt A., Karaali R., Mete B., Kuşkucu M.A., Aygün G., Keskindemirci Y. (2021). Clinical Characteristics of Mild-Moderate COVID-19 Patients and Risk Factors for the Development of Pneumonia. Mikrobiyol. Bul..

[34] Huang C., Wang Y., Li X., Ren L., Zhao J., Hu Y., Zhang L., Fan G., Xu J., Gu X. (2020). Clinical features of patients infected with 2019 novel coronavirus in Wuhan, China. Lancet.

[35] Guan W.J., Ni Z.Y., Hu Y., Liang W.H., Ou C.Q., He J.X., Liu L., Shan H., Lei C.L., Hui D.S. (2020). Clinical characteristics of coronavirus disease 2019 in China. N. Engl. J. Med..

[36] Goyal P., Choi J.J., Pinheiro L.C., Schenck E.J., Chen R., Jabri A., Satlin M.J., Campion T.R., Nahid M., Ringel J.B. (2020). Clinical characteristics of COVID-19 in New York city. N. Engl. J. Med..

[37] Wang D., Hu B., Hu C., Zhu F., Liu X., Zhang J., Wang B., Xiang H., Cheng Z., Xiong Y. (2020). Clinical characteristics of 138 hospitalized patients with 2019 novel coronavirus–infected pneumonia in Wuhan, China. JAMA.

[38] Guner R., Hasanoglu I., Kayaaslan B., Aypak A., Akinci E., Bodur H., Eser F., Kalem A.K., Kucuksahin O., Ates I. (2021). Comparing ICU admission rates of mild/moderate COVID-19 patients treated with hydroxychloroquine, favipiravir, and hydroxychloroquine plus favipiravir. J. Infect. Public Health.

[39] Khamis F., Al Naabi H., Al Lawati A., Ambusaidi Z., Al Sharji M., Al Barwani U., Pandak N., Al Balushi Z., Al Bahrani M., Al Salmi I. (2021). Randomized controlled open label trial on the use of favipiravir combined with inhaled interferon beta-1b in hospitalized patients with moderate to severe COVID-19 pneumonia. Int. J. Infect. Dis..

[40] Fujii S., Ibe Y., Ishigo T., Inamura H., Kunimoto Y., Fujiya Y., Kuronuma K., Nakata H., Fukudo M., Takahashi S. (2021). Early favipiravir treatment was associated with early defervescence in non-severe COVID-19 patients. J. Infect. Chemother..

[41] Chuah C.H., Chow T.S., Hor C.P., Cheng J.T., Ker H.B., Lee H.G., Lee K.S., Nordin N., Ng T.K., Zaid M. (2022). Efficacy of Early Treatment with Favipiravir on Disease Progression among High-Risk Patients with Coronavirus Disease 2019 (COVID-19): A Randomized, Open-Label Clinical Trial. Clin. Infect. Dis..

[42] McMahon J.H., Lau J.S., Coldham A., Roney J., Hagenauer M., Price S., Bryant M., Garlick J., Paterson A., Lee S.J. (2022). Favipiravir in early symptomatic COVID-19, a randomised placebo-controlled trial. EClinicalMedicine.

[43] Bosaeed M., Alharbi A., Mahmoud E., Alrehily S., Bahlaq M., Gaifer Z., Alturkistani H., Alhagan K., Alshahrani S., Tolbah A. (2022). Efficacy of favipiravir in adults with mild COVID-19: A randomized, double-blind, multicentre, placebo-controlled clinical trial. Clin. Microbiol. Infect..

[44] Venisse N., Peytavin G., Bouchet S., Gagnieu M.C., Garraffo R., Guilhaumou R., Solas C., Monitoring S.T.D., ANRS-AC43 Clinical Pharmacology Committee (2020). Concerns about pharmacokinetic (PK) and pharmacokinetic-pharmacodynamic (PK-PD) studies in the new therapeutic area of COVID-19 infection. Antiviral Res..

[45] Baker E.H., Gnjidic D., Kirkpatrick C.M.J., Pirmohamed M., Wright D.F.B., Zecharia A.Y. (2021). A call for the appropriate application of clinical pharmacological principles in the search for safe and efficacious COVID-19 (SARS-CoV-2) treatments. Br. J. Clin. Pharmacol..

[46] Gülhan R., Eryüksel E., Gülçebi İdriz Oğlu M., Çulpan Y., Toplu A., Kocakaya D., Tigen E., Ertürk Şengel B., Sili U., Olgun Yıldızeli Ş. (2022). Pharmacokinetic characterization of favipiravir in patients with COVID-19. Br. J. Clin. Pharmacol..

[47] Pertinez H., Rajoli R.K.R., Khoo S.H., Owen A. (2021). Pharmacokinetic modelling to estimate intracellular favipiravir ribofuranosyl-5’-triphosphate exposure to support posology for SARS-CoV-2. J. Antimicrob. Chemother..

[48] Madelain V., Nguyen T.H.T., Olivo A., De Lamballerie X., Guedj J., Taburet A.M., Mentré F. (2016). Ebola Virus Infection: Review of the Pharmacokinetic and Pharmacodynamic Properties of Drugs Considered for Testing in Human Efficacy Trials. Clin. Pharmacokinet..

[49] Chappell L., Horby P., Lim W.S., Emberson J.R., Mafham M., Bell J.L., Linsell L., Staplin N., Brightling C., Ustianowski A. (2021). Dexamethasone in hospitalized patients with COVID-19. N. Engl. J. Med..

[50] Van Paassen J., Vos J.S., Hoekstra E.M., Neumann K.M., Boot P.C., Arbous S.M. (2020). Corticosteroid use in COVID-19 patients: A systematic review and meta-analysis on clinical outcomes. Crit. Care.

[51] Doi Y., Hibino M., Hase R., Yamamoto M., Kasamatsu Y., Hirose M., Mutoh Y., Homma Y., Terada M., Ogawa T. (2020). A prospective, randomized, open-label trial of early versus late favipiravir therapy in hospitalized patients with COVID-19. Antimicrob. Agents Chemother..

[52] Ye J. (2020). The Role of Health Technology and Informatics in a Global Public Health Emergency: Practices and Implications From the COVID-19 Pandemic. JMIR Med. Inform..

[53] Monaghesh E., Hajizadeh A. (2020). The role of telehealth during COVID-19 outbreak: A systematic review based on current evidence. BMC Public Health.

